# Supplementary material for: Association of the risk of obstructive sleep apnoea with the severity of COVID-19
Source: PLoS One. 2023 Jul 18;18(7):e0284063. doi: 10.1371/journal.pone.0284063 (PMC10353795; doi:10.1371/journal.pone.0284063)
Supplement: Attachment — Submitted filename: response_to_reviewers.docx [file pone.0284063.s001.docx]

**Dear Editor-in-Chief,**

Re: *Revision and resubmission of manuscript no:* **PONE-D-22-31274**

Thank you for the opportunity to resubmit our revised manuscript. We have carefully read the reviewers' remarks and suggestions and have modified the manuscript accordingly. The changes in the revised manuscript are in red font.

We have responded to all comments and suggestions in a point-by-point manner immediately below this letter, where the reviewers' comments are in black and our responses are in red.

“The authors received no specific funding for this work.” The statistical analysis is available in OSF repository

DOI 10.17605/OSF.IO/7VUTXWe hope you will find our responses satisfactory. We believe that our research shows novel findings, and we look forward to working with you and the reviewers to move this manuscript closer to publication in *PLOS ONE*.

We would be glad to respond to any further questions and comments you may have, and we look forward to hearing from you.

Yours sincerely,

Arish Nissim, MD

Senior Physician

Pulmonary Institute

Shaare Zedek Medical Center

Jerusalem

Cell: (972)-(0)50-7163003

[nissim.arish@gmail.com](mailto:nissim.arish@gmail.com)

[narish@szmc.org.il](mailto:narish@szmc.org.il)

1. a) as stated by the Authors, two different COVID waves were considered. Were there any differences? and what about vaccination?

a. Thank you for these important questions. We did not observe any difference between the two COVID-19 waves. The COVID-19 vaccinations were not yet available at the time of the first two waves, so we could not assess their effect.

b) the risk of OSA was assessed. We suggest to delete paragraphs describing OSA and its physiopatology since no enrolled patient had a diagnosis of OSA?

b. Thank you for pointing this out. Indeed, the main limitation of our study is that the patients were not formally diagnosed with OSA with a sleep study; however, they filled out the Berlin questionnaire, which is used to screen for and evaluate the risk of OSA. Since our study aimed to assess the correlation of OSA with COVID-19 severity, we would like to retain the paragraph regarding OSA and its physiopathology.

c) we suggest to discuss to limits (if any) of the assessment of the "risk" of OSA
Follow-up data, if available, should be added. If not available, this should be addressed as a potential limitation.

c. Thank you for your suggestions. Unfortunately, we do not have follow-up data for the patients included in the study. Therefore, as requested by the reviewer, we have added this as a potential limitation of our study.

Please see the revised section below:

“Our study had some limitations. First, this retrospective observational study included a small sample of patients from a single medical center. In addition, patients with very severe diseases who died were not included in the study; thus, we could not obtain data regarding their risk for OSA. The data from these patients could have further contributed to the findings of this study. In addition, patients filled out the Berlin questionnaire retrospectively after developing COVID-19. This could have led to a bias, mainly in the fatigue-sleepiness section.

Second, the study was conducted in the first to third wave of the COVID-19 pandemic; thus, our findings may not apply to the Omicron era.

Third, we do not have long-term follow-up data for the patients, so we could not assess their long-term outcomes or confirm whether they had obstructive sleep apnea by completing a sleep test.”

2. Please include your full ethics statement in the 'Methods' section of your manuscript file. In your statement, please include the full name of the IRB or ethics committee who approved or waived your study, as well as whether or not you obtained informed written or verbal consent. If consent was waived for your study, please include this information in your statement as well.

Thank you for your comment. We had already included the full ethics statement, including the name of the ethics committee, in the Methods section (study design).

"The "Helsinki Ethics Committee" of the hospital approved this study [Reg: 0174-20-SZMC]. All the participants provided written informed consent."

Please let me know if you require more information.

. We note that the "Berlin Questionnaire© Sleep Apnea" in your submission contain copyrighted images. All PLOS content is published under the Creative Commons Attribution License (CC BY 4.0), which means that the manuscript, images, and Supporting Information files will be freely available online, and any third party is permitted to access, download, copy, distribute, and use these materials in any way, even commercially, with proper attribution.

Thank you for your remark. We have accordingly deleted the copyrighted image from the manuscript. The Berlin questionnaire is freely available online.
